# Expanding the Use of a Fluorogenic Method to Determine Activity and Mode of Action of *Bacillus thuringiensis* Bacteriocins Against Gram-Positive and Gram-Negative Bacteria

**DOI:** 10.1100/2012/503269

**Published:** 2012-08-01

**Authors:** Norma M. de la Fuente-Salcido, J. Eleazar Barboza-Corona, A. N. Espino Monzón, R. D. Pacheco Cano, N. Balagurusamy, Dennis K. Bideshi, Rubén Salcedo-Hernández

**Affiliations:** ^1^Escuela de Ciencias Biológicas, Universidad Autónoma de Coahuila, 27440 Torreón, COAH, Mexico; ^2^División Ciencias de la Vida, Departamento de Alimentos, Universidad de Guanajuato Campus Irapuato-Salamanca, 36500 Irapuato, GTO, Mexico; ^3^Department of Natural and Mathematical Sciences, California Baptist University, 8432 Magnolia Avenue, Riverside, CA 92504, USA; ^4^Department of Entomology, University of California, Riverside, CA 92521, USA

## Abstract

Previously we described a rapid fluorogenic method to measure the activity of five bacteriocins produced by Mexican strains of *Bacillus thuringiensis* against *B. cereus* 183. Here we standardize this method to efficiently determine the activity of bacteriocins against both Gram-positive and Gram-negative bacteria. It was determined that the crucial parameter required to obtain reproducible results was the number of cells used in the assay, that is, *~*4 × 10^8^ cell/mL and *~*7 × 10^8^ cell/mL, respectively, for target Gram-positive and Gram-negative bacteria. Comparative analyses of the fluorogenic and traditional well-diffusion assays showed correlation coefficients of 0.88 to 0.99 and 0.83 to 0.99, respectively, for Gram-positive and Gram-negative bacteria. The fluorogenic method demonstrated that the five bacteriocins of *B. thuringiensis* have bacteriolytic and bacteriostatic activities against all microorganisms tested, including clinically significant bacteria such as *Listeria monocytogenes, Proteus vulgaris,* and *Shigella flexneri* reported previously to be resistant to the antimicrobials as determined using the well-diffusion protocol. These results demonstrate that the fluorogenic assay is a more sensitive, reliable, and rapid method when compared with the well-diffusion method and can easily be adapted in screening protocols for bacteriocin production by other microorganisms.

## 1. Introduction

Antimicrobial peptides are biomolecules synthesized by both eukaryotic and prokaryotic organisms. They are involved in multiple functions, such as protection against the attack by microorganisms, endotoxin neutralization, chemotactic and immunomodulating activities, induction of angiogenesis, and wound repair [1]. Antimicrobial peptides produced by bacteria are called bacteriocins [2]. Among the most studied bacteriocins are those synthesized by *Lactobacillus* species. However, the screening of new microorganisms that produce antimicrobial peptides with biotechnological applied use has been expanded to other types of bacteria, including *Bacillus *spp. In this regard, *Bacillus thuringiensis*, one of the most important bioinsecticide used worldwide, synthesizes different types of bacteriocins that elaborate inhibitory activity against a rich diversity of bacteria including human pathogens, and those involved in food spoilage, foulbrood disease of honeybees, and crop diseases [3–11]. In particular, Morricin 269, Kurstacin 287, Kenyacin 404, Entomocin 420, and Tolworthcin 524 are bacteriocins produced by Mexican strains of *B. thuringiensis* that are active in a wide range of temperatures and pH against Gram-positive and Gram-negative bacteria, and thus could be of applied interest to industry [10]. 

As the search for viable and stable bacteriocins for use in the food and medicinal industries is expanding, developing new rapid and sensitive protocols could facilitate screening and identification of microbes that produce these peptides. Methods for detecting bacteriocin producers are based on enzymatic and nonenzymatic procedures, the former being a more sensitive technique [12–14]. Recently, an innovative and rapid fluorogenic method for detecting bacteriocin activity based on berberine fluorescence following its influx into compromised cells of *Bacillus cereus *183 was described [15]. Using this fluorogenic method, bacteriocin activity was accurately determined within one hour which was much more rapid than conventional methods that are tedious and time consuming, especially methods employing well-diffusion assays [13, 14]. 

In order to expand the applied use of the fluorogenic method, here we standardize the conditions to evaluate the antimicrobial activity of the five bacteriocins synthesized by *B. thuringiensis* against both Gram-positive and Gram-negative pathogenic bacteria and also determine the underlying modes of action of these antimicrobial peptides. The method is sensitive, rapid, and reliable and can be easily adapted to screen and identify bacteriocin activities of other microbes. In addition, we show that the bacteriocins produced by *B. thuringiensis* elicit both bacteriolytic and bacteriostatic effects against target microorganisms of clinical significance.

## 2. Material and Methods

### 2.1. Bacterial Strains 

Bacteriocin-producing strains, used in this study, were *Bacillus thuringiensis* subsp. *morrisoni* (LBIT 269), *B. thuringiensis* subsp. *kurstaki* (LBIT 287), *B. thuringiensis* subsp *kenyae* (LBIT 404), *B. thuringiensis* subsp. *entomocidus* (LBIT 420), and *B. thuringiensis* subsp. *tolworthi* (LBIT 524) obtained from a native bacterial stock collection held at CINVESTAV, Campus Guanajuato, Mexico. These strains synthesize Morricin 269, Kurstacin 287, Kenyacin 404, Entomocin 420, and Tolworthcin 524, respectively [11]. *Bacillus cereus* 183 was obtained from a collection of *Bacillus *strains maintained in the International Entomopathogenic *Bacillus* Centre, Institute Pasteur in Paris, France and was used as the indicator bacterium for the determination of bacteriocin activity in well diffusion method. Activity of the bacteriocins were determined against Gram-positive (*Staphylococcus xylosus* ATCC 700404, *S. aureus* ATCC 25923, *Bacillus cereus* 183, *B. subtilis* ATCC 6633, *Listeria monocytogenes, Micrococcus species* ATCC 700405, *Streptococcus agalactiae, Streptococcus pyogenes,* and *Enterococcus faecalis* ATCC 10541) and Gram-negative bacteria (*Pseudomonas aeruginosa* ATCC 27853, *Proteus vulgaris* ATCC 13315, *Escherichia coli* ATCC 25932, *Klebsiella pneumoniae, Enterobacter cloacae* ATCC 13047, **Salmonella** *sp*., *Salmonella typhimurium* ATCC 14022, *Serratia marcescens Nima, Serratia marcescens WF, Shigella flexneri, and Shigella sonnei*).

### 2.2. Bacteriocin Production 

Each producer strain was cultivated in tryptic soy broth (TSB) for the time where the highest bacteriocin activity was detected in kinetic studies as previously described [10]. Cultures were centrifuged at 10,000 ×g for 15 min, and the supernatant was filtered through 0.20 mm filter. Supernatants were concentrated with ammonium sulfate to 80% saturation and precipitated at 4°C with constant stirring overnight. Precipitated proteins were pelleted by centrifugation at 16,000 ×g for 30 min at 4°C, resuspended in 100 mM phosphate buffer (pH 7.0), dialyzed overnight against the same buffer using a mini-dialysis kit with a 1 kDa cutoff (Amersham Biosciences), and stored at −20°C for subsequent studies.

### 2.3. Adjustment of Bacterial Concentration 

Depending on the strain tested, bacteria were grown overnight in TSB, Brain Heart Infusion (BHI), Luria-Bertani Broth (LB), Tetrathionate Broth (TB), Nutrient Broth (NB), de Man, Rogosa and Sharp Broth (MRSB), at 28 or 37°C and 200 rpm (see Table 2). One volume of each culture was mixed with 4 volumes of TSB and incubated at 28 or 37°C, for 2 h at 200 rpm. Then cell pellets were obtained by centrifugation at 10,000 × g for 15 min and resuspended in 50 mM phosphate buffer with 5% (v/v) glycerol (PBG) to adjust the bacterial concentration to ~4×10^8^ cell/mL and ~7×10^8^ cell/mL, respectively, for Gram-positive and Gram-negative bacteria [15]. 

### 2.4. Evaluation of Antibacterial Activity by Fluorogenic Method 

20 *μ*L with ~4×10^8^ cell/mL or ~7×10^8^ cell/mL were mixed with different volumes (10, 20, 30, 40, 50 *μ*L) of each bacteriocin, 24 *μ*M of berberine sulfate (Sigma), and 50 mM PBG to reach a volume of 1000 *μ*L. All samples were incubated at room temperature and fluorescence was determined in a Turner fluorometer (model 450; 340-nm interference filter and 415 nm cut filter). Triplicate fluorescence assays were performed and average values were plotted against bacteriocin concentrations. In addition, bacteria tested with each bacteriocin and berberine were observed by fluorescence microscopy with a ND filter (Eclipse E200, Nikon). Bacteria treated only with bacteriocins were stained with 0.005% (w/v) amido black 10B (BioRad) in 50% (v/v) ethanol and observed under light microscopy (Axio Imager A1, Carl Zeiss). 

### 2.5. Evaluation of Antibacterial Activity by Well-Diffusion Assay 

Concomitant with the fluorogenic assays, 25 mL of TSB with soft agar 0.7% (wt/vol) was mixed with 50 *μ*L (~1 × 10^9^ cell/mL) of each tested culture and plated. The same volumes (10, 20, 30, 40, 50 *μ*L) of bacteriocins used in the fluorescence were added to wells, 7 mm in diameter, and plates were incubated for 12 h at 4°C to allow the diffusion of the samples, followed by an additional incubation at 28 or 37°C for one day before diameters of zones of inhibition were measured. One unit (U) of activity was defined as 1 mm^2^ of the zone of inhibition of growth of the indicator bacterium [10]. Finally, fluorescence average values were plotted against average of activity determined by well-diffusion method and the correlation between both methodologies was established [16, 17].

### 2.6. Effect of Bacteriocins on the Growth of Bacterial Cultures 

*B. cereus and *Salmonella** *sp*. were selected to study the effect of bacteriocin on bacterial cultures. Both microorganisms were grown overnight and ~1 × 10^9^ cells/mL was inoculated in 100 mL of fresh broth (TSB). *B. cereus and *Salmonella** *sp.* cultures were incubated for ~4 and 3 hr at 28°C or 37°C, respectively, with constant stirring at 180 rpm in an orbital shaking incubator (Shel Lab, Cornelius, OR, USA), to reach the middle of their logarithmic-phase (log phase), and then ~3000 U of bacteriocin was added to the flask. Units (U) were determined with the well-diffusion method using *B. cereus* as indicator bacterium as shown previously [10]. The optical density (OD_660 nm_) was monitored at 5, 15, 45, and 60 minutes in triplicate assays using a Smartspec 3000 Spectrophotometer (Bio-Rad). Additionally, the number of viable cells (CFU mL^−1^) was determined by total viable count using samples serially diluted with saline solution (0.85% w/v) and plated onto plate count agar during 24 h at 28 or 37°C [6, 18]. In both cases, duplicate cultures were grown without adding bacteriocins and used as controls.

## 3. Results

### 3.1. Standardization of the Fluorogenic Method with a Gram-Negative Bacterium (*Salmonella *sp*.*) 

The fluorogenic method using berberine was previously standardized with a Gram-positive bacterium (i.e., *B. cereus*) [15]. In order to expand the practical application of this method to evaluate the bacteriocin activity to Gram-negative bacteria, we tested the susceptibility of *Salmonella sp. *using the fluorogenic method. We determined that conditions were similar to that reported in the *B. cereus *assay [17]. However, the crucial difference to obtain reproducible results was the number of cells used in the assay. For assays with *Salmonella sp.*, it was necessary to use ~7 × 10^8^ cell/mL (optical density of ~1.5), compared to assays with *B. cereus, *where ~4 ×10^8^ cell/mL (optical density of 0.5 to 1.0) [15] were required to obtain optimal and reproducible results. 

**Figure 1:**
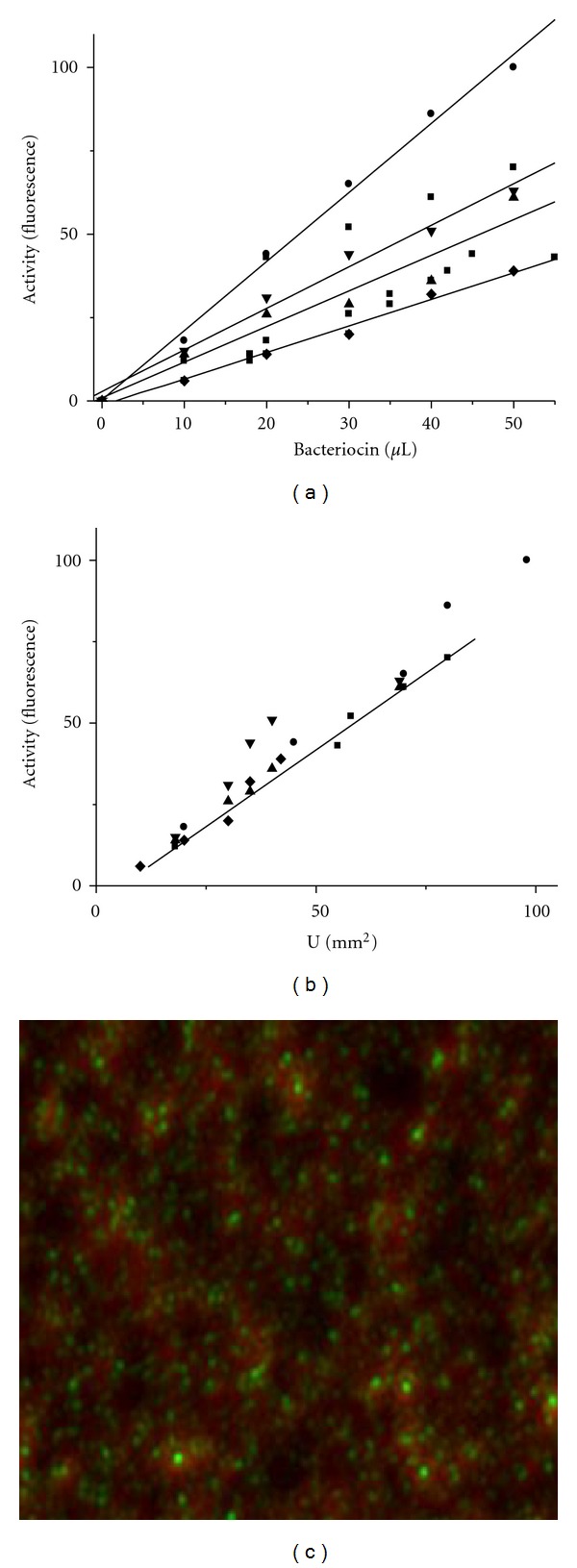
(a) Fluorescence emitted by berberine sulfate upon influx into *Salmonella sp*. treated with different concentrations of bacteriocins produced by* B. thuringiensis*. (b) Correlation (*r* = 0.94) between the bacteriocin activity in mm^2^ of inhibition area and fluorescence. Morricin 269 (■); Kurstacin 287 (●); Kenyacin 404 (▲); Entomocin 420 (*▼*); Tolworthcin 524 (◆). (c) Fluorescence emitted by berberine sulfate inside **Salmonella** *sp*. after the effect on cytoplasmic membrane induced by bacteriocin Kurstacin 287.

In previous work [12] it was observed that bacteriocins tested against *B. cereus *183 showed a linear increment of fluorescence with bacteriocin concentration, that is, the higher bacteriocin concentration, the higher fluorescence. Similar behavior was observed with *Salmonella sp*., using up to 50 *μ*L of each crude bacteriocin preparation. Correlation coefficients (*r*) of each bacteriocin assayed separately were 0.96 for Kenyacin 404, 0.97 for Morricin 269, and 0.99 for Kurstacin 287, Entomocin 420, and Tolworthcin 524 (Figure 1(a)). When bacteriocins were evaluated against *Salmonella sp*. and activity was measured as the area of inhibition (mm^2^) and also as the fluorescence emitted by berberine, a correlation of 0.94 was observed, suggesting that the fluorogenic method could be efficiently substituted for the well-diffusion method against Gram-negative bacteria. Accordingly with the assay against *Salmonella sp*., the maximum inhibition area of 100 mm^2^ that correspond to a relative fluorescence of 100 still gave a linear response (Figure 1(b)). 

### 3.2. Correlation between the Fluorogenic Method and the Well-Diffusion Assay 

In order to confirm that bacteriocin activity determined by fluorescence correlates with the standard agar diffusion methods not only with *B. cereus *183 [15] and *Salmonella sp*. but also with other Gram-negative and Gram-positive bacteria, comparable experiments were performed in triplicate using the same conditions. When bacteriocins were tested against Gram-positive bacteria, fluorogenic and well-diffusion methods showed correlation coefficients (*r*) of 0.88 to 0.99. Likewise, they showed correlations of 0.83 to 0.99 when bacteriocins were assayed against Gram-negative bacteria (Table 1). 

### 3.3. Susceptibility of Bacteria to Bacteriocins Tested with the Fluorogenic Method

The results described above confirmed the utility of the fluorogenic method to evaluate the inhibitory effect of bacteriocins against bacteria in a short period of time. Thus, the method was used to determine bacteriocin susceptibilities against Gram-negative bacteria such as *P. aeruginosa, P. vulgaris, E. coli, K. pneumoniae, E. cloacae, Salmonella sp., S. typhimurium, S. marcescens Nima, S. marcescens wf, Shigella flexneri, *and* Shigella sonnei* and also of Gram-positive, *S. xylosus, S. aureus, B. cereus 183, B. subtilis, L. monocytogenes, Micrococcus sp., *Str*.agalactiae, *Str*.pyogenes,* and* E. faecalis* (Table 2).

### 3.4. Pore-Forming Activity 

When cells of **Salmonella** *sp*. were treated with bacteriocins, the cellular membrane was damaged allowing the entrance of berberine (a nonpermeable alkaloid) that fluoresces inside the cells (Figure 1(c)). It was observed that the higher bacteriocin concentration the higher fluorescence was emitted, indicating an increment in the cellular damage (Figure 1(a)). Similar results were recorded when *B. cereus *183 was treated with  bacteriocins-berberine and observed under fluorescence microscopy (data not shown). Likewise, an increment in the bacteriocin concentrations generated an augmentation of the fluorescence emitted by *B. cereus *[15].

### 3.5. Mode of Action of Bacteriocins Synthesized by Bacillus thuringiensis 

The five bacteriocins of *B. thuringiensis* were added in the middle of the logarithmic phase of the growth curve of a Gram-positive (*B. cereus *183) and Gram-negative (**Salmonella** *sp*.) bacterium, and the effect on the optical density and the bacterial viability was evaluated. 

**Figure 2:**
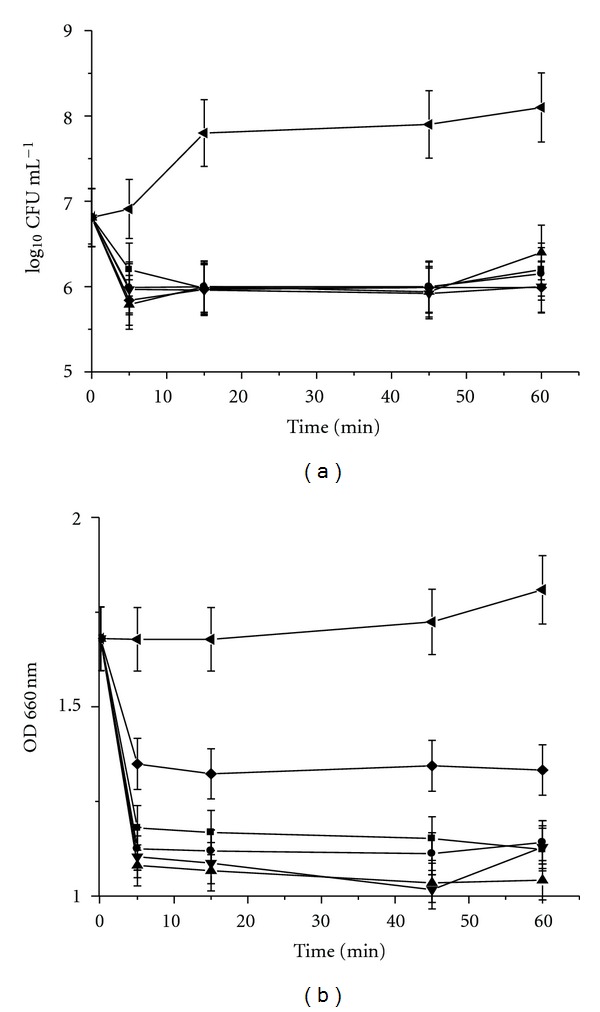
Effect of bacteriocins produced by *B. thuringiensis* on the growth of *B. cereus* 183. (a) Log_10_ cfu mL^−1^. (b) Optical density measured at 660 nm. The five bacteriocins were added in the middle of the logarithmic phase of growth curve (~4 h) of *B. cereus* (indicator strain) used as control (without bacteriocins). *B. cereus* 183 (◂); Morricin 269 (■); Kurstacin 287 (●); Kenyacin 404 (▲), Entomocin 420 (*▼*); Tolworthcin 524 (◆).

**Figure 3:**
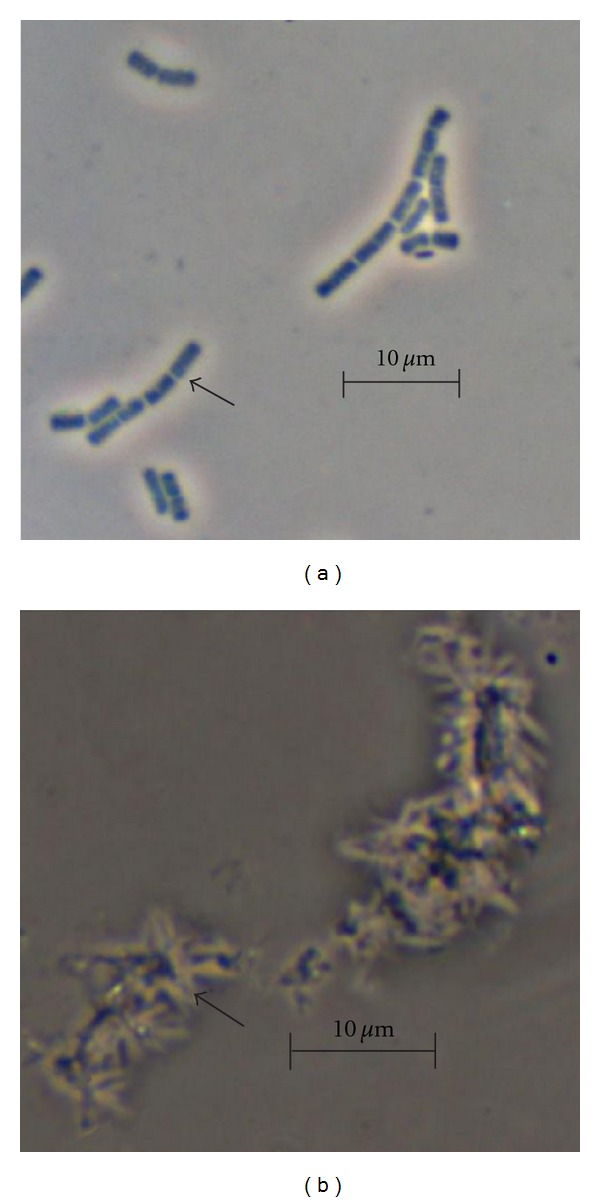
Micrographs of *B. cereus* 183 cells treated with Kurstacin 287. Samples were treated with bacteriocin, stained with amido black 10B (BioRad) for 5 min and observed under light microscopy. (a) *B. cereus* 183 cells without bacteriocin (control), (b) *B. cereus* 183 cells with Kurstacin 287. Black arrow in (a) and (b) show cells of *B. cereus* not damaged or lysed, respectively.

When bacteriocins were added to *B. cereus, *a decrement of ~5–12-fold in the indicator strain viable cell number was observed after 5 min of exposure, when compared with cells not treated with the antimicrobial peptides. Similarly, a diminution in the optical density was detected at 5 min of incubation. The decrease in the colony forming unit (CFU) and the optical density were maintained after a longer period of time (i.e., 60 min) (Figure 2). These results suggested that the five bacteriocins of *B. thuringiensis *exhibited a bacteriolytic effect against a Gram-positive bacterium. Bacteriolytic effect was confirmed when *B. cereus *was treated with Kurstacin 287 and observed under light microscopy after 5 min of incubation. A drastic effect occurred, where cells lost membrane integrity and lysed (Figure 3). A similar effect was observed with the other bacteriocins of *B. thuringiensis *(data not shown).

**Figure 4:**
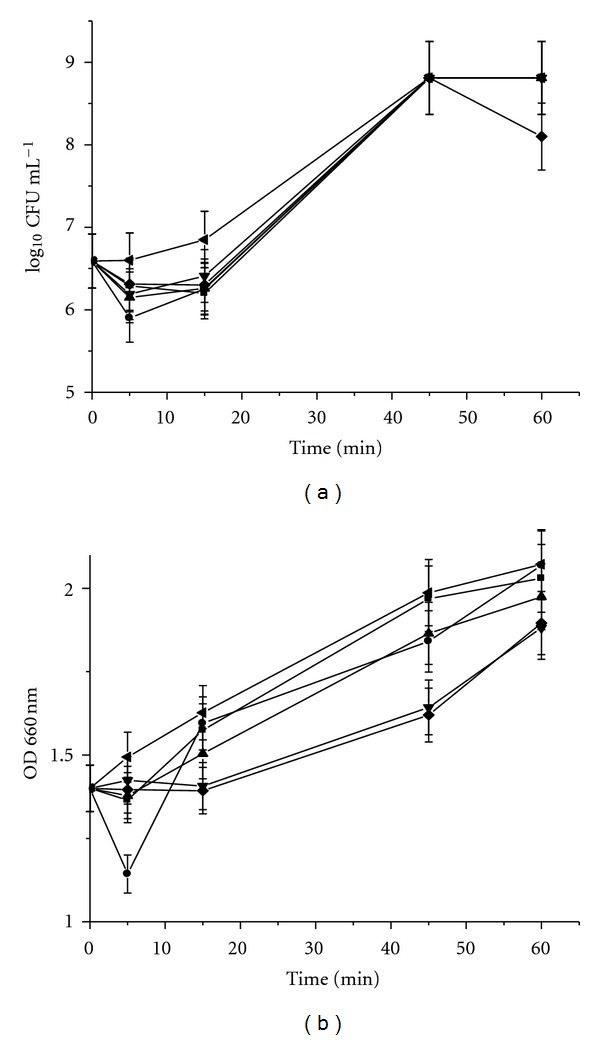
Effect of bacteriocins produced by *B. thuringiensis* on the growth of **Salmonella** *sp.* (a) Log_10_ cfu mL^−1^ (b) optical density measured at 660 nm. The five bacteriocins were added in the middle of the logarithmic phase of growth curve (~3 h) of **Salmonella** *sp*. culture (indicator strain) used as control (without bacteriocins). **Salmonella** *sp*. (◂); Morricin 269 (■); Kurstacin 287 (●); Kenyacin 404 (▲), Entomocin 420 (*▼*); Tolworthcin 524 (◆).

Addition of the five bacteriocins to cultures of **Salmonella** *sp*. showed that this bacterium was less susceptible than *B. cereus *183 to the antimicrobial peptides. After 5 min of the addition of bacteriocins, a decrement in both viable cells and optical density was observed. However, over longer periods of incubation, almost parallel increases in both viable cell count and optical density was observed, although values were consistently lower than those of the control cells not treated with bacteriocins (Figure 4). These results suggested that bacteriocins of *B. thuringiensis *produced a low bacteriostatic effect against a Gram-negative bacterium.

## 4. Discussion

We initially standardized the fluorogenic method to determine the antibacterial activity of bacteriocins of **B. thuringiensis ** against *B. cereus* 183 and also evaluated the utility of the methodology to determine the susceptibility of other Gram-positive bacterium (i.e., *Listeria innocua*) to Nisin and Pediocin [12]. Using similar parameters in that study, we were unable to obtain reproducible results against Gram-negative bacteria. Our present study shows that in order to expand this technology to include screenings against Gram-negative bacteria a significant increase in the number of cells was required, using **Salmonella** *sp*. as the model target. Although we do not have a specific reason why this is so, this observation is possibly related to the difference in cellular envelope composition between Gram-positive and Gram-negative bacteria [19, 20]. Gram-negative bacteria have a lipopolysaccharide coat surrounding the peptidoglycan layer that could prolong penetration of berberine into the bacteriocin-treated cell. Thus reproducible collective fluorescence signals could be obtained with more cells present in the assay as compared to lower levels when fewer cells are used. It should be noted that similar increment in fluorescence was obtained with the other Gram-negative bacteria in assays with higher cell density.

Although bacteriocins vary in their spectrum of activity, mode of action, molecular weight, genetic origin, and biochemical properties [21], bacteriocins synthesized by a Gram-positive bacterium typically have activity only against other Gram-positive species[21–24]. We previously determined by the well-diffusion method that bacteriocins produced by Mexican strains of *B. thuringiensis *have inhibitory effect against both Gram-positive and Gram-negative bacteria [25–29], which was confirmed in this work by the fluorogenic method. In this regard, antibacterial activity of bacteriocins as determined by the fluorogenic and the well-diffusion methods against Gram-positive and Gram-negative bacteria showed a high level of correlation coefficients, indicating that the flourogenic method can substitute accurately for the well-diffusion method. In addition, because of the reliability of the fluorogenic methodology, we were able to detect the susceptibility of a different class of bacteria to bacteriocins of *B. thuringiensis *not observed by the well-diffusion method. For example, we reported that the five bacteriocins of *B. thuringiensis *are not toxic to *L. monocytogenes* [10], one of the most virulent foodborne pathogen that is the causative agent of the listeriosis. Here, using the fluorogenic method, we demonstrated susceptibility of this bacterium to all bacteriocins tested (Table 2).  Likewise, *P. vulgaris *and *Shigella flexneri, *etiological agents of urinary tract infections and diarrhea in humans, respectively, showed no susceptibility to at least two of the bacteriocins of *B. thuringiensis *as determined by the well-diffusion assay [25], but the antibacterial effect of the bacteriocins was observed with the fluorogenic method (Table 2). Interestingly, we detected antibacterial activity with both methods against *Streptococcus agalactiae*, one of the most important etiologic agents of mastitis in cattle [30]. To our knowledge, this is the first report on the effect of bacteriocins synthesized by *B. thuringiensis* against this bacterium. We previously report the effect of these bacteriocins against *Staphylococcus aureus*, a common human pathogen that is also associated with bovine mastitis [31]. These results suggest the potential applied use of these bacteriocins to control these two etiological agents of mastitis in animals.

The influx of berberine into bacteriocin-treated cells causes the molecule to fluoresce (Figure 1) upon interaction with intracellular components, including DNA and glycosaminoglycans [17, 32, 33]. The mechanisms of action of the bacteriocins produced by *B. thuringiensis* are not known. In particular, data on their interaction with structural components of lipopolysaccharide and peptidoglycan layers are lacking. Although we do not know the amino acid sequence of bacteriocins of this study, it is probably that these antimicrobial peptides have positive charges that could interact with the negative charge of the anionic phospholipids, or with the phosphate groups of lipopolysaccharide located on the outer membrane layer of Gram-negative, and also with the teichoic acids of Gram-positive bacteria by electrostatic forces. Such interactions are known to subsequently change the permeability of cellular membrane by a pore formation [34–37]. The pore formation is widespread between cationic bacteriocins such as Thuricin S of* B. thuringiensis* subsp. *entomocidus* HD198 [38]. Additionally, it has been observed that bacteriocins can produce different pore sizes in cellular membranes that could be an important factor on the mode of action of the antimicrobial peptide [39]. Regardless, data obtained in this work indicate that bacteriocins of *B. thuringiensis* have both bacteriolytic and bacteriostatic effects, respectively, against Gram-positive and Gram-negative bacteria. It is evident that the absence (i.e., Gram-positive bacteria) or presence (i.e., Gram-negative bacteria) of an outer membrane layer is crucial to define the mode of action (Figures 2 and 3). A dual effect has also been reported with Thuricin 17, Entomocin 110 and Thuricin 7 that have bactericidal-bacteriostatic and bactericidal-bacteriolytic action [7, 11, 18]. Therefore, the presence of an outer lipopolysaccharide membrane of Gram-negative bacteria could be a significant factor that limits efficient intracellular localization of the *B. thuringiensis* bacteriocins studied here. 

In conclusion, we have demonstrated that the fluorogenic method is a rapid protocol to determine the antibacterial activity of bacteriocins of *B. thuringiensis* against Gram-positive and Gram-negative bacteria, and that the method is a reliable substitute for the traditional well-diffusion method. Moreover, the method could be easily adapted in large-scale screenings of microorganism that produce bacteriocins, and perhaps other antimicrobial agents that disrupt cell wall structures. Our future efforts will focus on purification of bacteriocins of *B. thuringiensis *used in this work, cloning their corresponding genes, and designing methods to mass produce these peptides for both applied and basic biochemical studies, including the mechanism of action. 

## Figures and Tables

**Table 1:** Correlation coefficient between the fluorogenic method and well diffusion assay.

Strain			Correlation		
	Morricin 269	Kurstacin 287	Kenyacin 404	Entomocin 420	Tolworthcin 524
Gram-negative					
*Proteus vulgaris*	0.96	0.95	0.89	0.95	0.95
*Klebsiella pneumoniae*	0.98	0.99	0.88	0.99	0.91
*Salmonella sp.*	0.97	0.99	0.96	0.99	0.99
*Shigella flexneri*	0.95	0.95	0.93	0.89	0.95
*Shigella sonnei*	0.95	0.99	0.99	0.93	0.93
Gram-positive					
* Bacillus cereus *183	0.99	0.99	0.99	0.99	0.97
*Bacillus subtilis*	0.87	0.90	0.94	0.94	0.83
*Listeria monocytogenes*	0.92	0.93	0.94	0.96	0.98
*Enterococcus faecalis*	0.94	0.94	0.99	0.95	0.99

**Table 2:** Inhibitory activity (UF) of bacteriocins synthesized by* B. thuringiensis* determined by fluorogenic method.

Strain	Bacteriocin activity					
	Morricin 269	Kurstacin 287	Kenyacin 404	Entomocin 420	Tolworthcin 524	Culture media^∗^, ^°^C^a^
Gram-negative						
*Pseudomonas aeruginosa *	74	109	120	81	106	TSB
*Proteus vulgaris*	110	78	67	93	52	TSB/BHI
*Escherichia coli*	77	97	120	68	100	TSB/LB
*Streptococcus pyogenes*	80	109	123	79	109	TSB
*Klebsiella pneumoniae*	76	96	114	82	115	TSB
*Enterobacter cloacae*	101	93	110	67	101	TSB
*Salmonella sp.*	70	126	61	63	39	TSB/TB
*Salmonella typhimurium*	71	105	128	82	121	TSB/TB
*Serratia marcescens Nima*	114	97	110	70	108	TSB
*Serratia marcescens wf*	95	93	110	75	100	TSB
*Shigella flexneri*	80	110	131	118	110	TSB/TB
*Shigella sonnei*	70	84	105	66	98	TSB/TB
Gram-positive						
*Staphylococcus xylosus *	87	77	98	133	100	NB
*Staphylococcus aureus *	78	90	118	76	95	NB
* Bacillus cereus *183	125	89	64	96	82^a^	TSB/LB^a^
*Bacillus subtilis*	72	106	129	83	110^a^	TSB/LB^a^
*Listeria monocytogenes*	103	96	110	67	96	NB
*Micrococcus sp.*	111	100	122	71	106^a^	TSB/NB^a^
*Streptococcus agalactiae*	106	90	73	91	50	NB/BHI
*Enterococcus faecalis*	109	98	125	73	104	MRSB

^∗^ Culture media—TSB: Trypticase Soy Broth; BHI: Brain Heart Infusion; LB: Luria-Bertani Broth; TB: Tetrathionate Broth; NB: Nutrient Broth; MRSB: de Man, Rogosa and Sharp Broth.

^a^All bacteria were incubated at 37^°^C, except *B. cereus* 183, *B. subtilis,* and *Micrococcus sp., *that were cultivated at 28^°^C.
